# Activation of the Met kinase confers acquired drug resistance in FGFR-targeted lung cancer therapy

**DOI:** 10.1038/oncsis.2016.48

**Published:** 2016-07-18

**Authors:** S-M Kim, H Kim, M R Yun, H N Kang, K-H Pyo, H J Park, J M Lee, H M Choi, P Ellinghaus, M Ocker, S Paik, H R Kim, B C Cho

**Affiliations:** 1 JE-UK Institute for Cancer Research, JEUK Co., Ltd., Gumi, Kyungbuk, Korea; 2 Bayer Pharma AG, Global Drug Discovery, Wuppertal, Germany; 3 Division of Pathology NSABP, Pittsburgh, PA, USA; 4 Severance Biomedical Science Institute, Yonsei University College of Medicine, Seoul, Korea; 5 Division of Medical Oncology, Department of Internal Medicine, Yonsei Cancer Center, Yonsei University College of Medicine, Seoul, Korea

## Abstract

Aberrant fibroblast growth factor receptor (FGFR) activation/expression is a common feature in lung cancer (LC). In this study, we evaluated the antitumor activity of and the mechanisms underlying acquired resistance to two potent selective FGFR inhibitors, AZD4547 and BAY116387, in LC cell lines. The antitumor activity of AZD4547 and BAY1163877 was screened in 24 LC cell lines, including 5 with *FGFR1* amplification. Two cell lines containing *FGFR1* amplifications, H1581 and DMS114, were sensitive to FGFR inhibitors (IC_50_<250 nm). Clones of *FGFR1*-amplified H1581 cells resistant to AZD4547 or BAY116387 (H1581AR and H1581BR cells, respectively) were established. Receptor tyrosine kinase (RTK) array and immunoblotting analyses showed strong overexpression and activation of Met in H1581AR/BR cells, compared with that in the parental cells. Gene set enrichment analysis against the Kyoto Encyclopedia of Genes and Genomes (KEGG) database showed that cytokine–cytokine receptor interaction pathways were significantly enriched in H1581AR/BR cells, with Met contributing significantly to the core enrichment. Genomic DNA quantitative PCR and fluorescent *in situ* hybridization analyses showed *MET* amplification in H1581AR, but not in H1581BR, cells. Met amplification drives acquired resistance to AZD4547 in H1581AR cells by activating ErbB3. Combination treatment with FGFR inhibitors and an anaplastic lymphoma kinase (ALK)/Met inhibitor, crizotinib, or Met-specific short interfering RNA (siRNA) synergistically inhibited cell proliferation in both H1581AR and H1581BR cells. Conversely, ectopic expression of Met in H1581 cells conferred resistance to AZD4547 and BAY1163877. Acquired resistance to FGFR inhibitors not only altered cellular morphology, but also promoted migration and invasion of resistant clones, in part by inducing epithelial-to-mesenchymal transition. Taken together, our data suggest that Met activation is sufficient to bypass dependency on FGFR signaling. Concurrent inhibition of the Met and FGFR pathways may have synergistic clinical benefits when targeting FGFR-dependent LC.

## Introduction

Lung cancer (LC) is the leading cause of cancer-related mortality worldwide.^1^ Recently, there has been considerable advances of molecularly-targeted therapies in LC patients; epidermal growth factor receptor (EGFR) tyrosine kinase inhibitors (TKIs), including gefitinib, erlotinib and afatinib, are utilized in patients with EGFR mutations, while the anaplastic lymphoma kinase (ALK) inhibitor, crizotinib, is employed in those with ALK rearrangement.^2^ One of the newly recognized molecular targets in LC is FGFR. Our groups and others have already reported *FGFR1* amplification in squamous cell carcinoma, a major histologic subtype of LC, at a frequency of 13–22%.^3, 4, 5, 6^

Fibroblast growth factors receptor (FGFR) gene amplification and overexpression is a common alteration and a potential drug target in LC.^7^ Aberrant FGF signaling can promote tumor development by directly driving cancer cell survival, motility, invasiveness, proliferation, epithelial-to-mesenchymal transition (EMT) and angiogenesis.^8^ Activation of FGF signaling leads to phosphorylation of the bound fibroblast growth factor receptor substrate 2 (FRS2) and downstream activation of Ras/Raf/MAPK (mitogen-activated protein kinase), phosphoinositide 3-kinase (PI3K)/AKT and Janus kinase/signal transducer and activator of transcription (STAT) pathways.^9^

A number of FGFR-targeted agents are currently being developed in LC harboring FGFR alterations. The TKIs targeting the ATP-binding site of FGFRs can be classified into two different types; multi-target FGFR-TKIs such as PD173074, dovitinib and ponatinib, and highly-selective FGFR inhibitors such as AZD4547, BGJ398, LY287445 and BAY1163877.^9^ AZD4547 is a highly active pan-FGFR selective inhibitor that was shown to block FGFR signaling and proliferation in cancer cell lines with deregulated FGFR expression.^10^ Recently, a phase Ib trial (NCT00979134) assessing the efficacy and safety of AZD4547 in advanced squamous cell LC harboring *FGFR1* amplification reported overall response rate of 8%.^11^ BAY1163877 selectively inhibits FGFR1–3 kinase activity, phosphorylation of downstream signaling molecules and proliferation of various cancer cell lines.^12^ A phase I clinical trial (NCT01976741) is underway to test the safety and preliminary antitumor activity of BAY1163877.^13^

All effective molecularly-targeted cancer therapies are hampered by acquired drug resistance. Reactivation of the target through a secondary mutation, activation of upstream or downstream effectors and/or activation of a bypass oncoprotein have been implicated in acquired drug resistance.^14^ The bypass resistance mechanism results in the activation of a critical downstream signaling effector through a parallel mechanism that is indifferent to the kinase-directed therapy, which has been well described in EGFR-mutant LC.^14^ Here, aberrant reactivation of PI3K/AKT signaling in the presence of gefitinib (an EGFR-TKI) can occur as a result of Met activation through either *MET* amplification or by its ligand hepatocyte growth factor.^15, 16^ Notably, *MET* amplification causes resistance to gefitinib by driving ErbB3-dependent activation of PI3K.^17^ In addition to drug resistance, Met activation promotes cancer progression, metastasis, cancer cell migration and angiogenesis.^15^

Recent reports have suggested a signaling crosstalk between FGFR and Met. In Met-dependent cell lines such as MKN45 (gastric cancer) and EBC-1 (LC), FGFR is a key regulator of resistance to a Met-targeting antibody.^18^ In acute myeloid leukemia, FGFR1 activity is required for the compensatory upregulation of hepatocyte growth factor in response to Met inhibition.^19^ Furthermore, hepatocyte growth factor secretion compensates for cancer cell growth inhibition by BGJ398, a selective FGFR inhibitor.^20^

In this study, we evaluated the activity of selective FGFR inhibitors (AZD4547 and BAY116387) and the mechanisms of acquired resistance to these agents in LC. We established an acquired resistance model *in vitro*, in which an FGFR-TKI-sensitive cell line, H1581, was subjected to long-term treatment with AZD4547 and BAY1163877. We confirmed activation of the Met pathway in cells that had acquired resistance to AZD4547 and BAY1163877 (H1581AR and H1581BR). We provide evidence in support of the hypothesis that Met activation is sufficient to bypass dependency on FGFR signaling. Our data suggest that concurrent inhibition of these two pathways may be desirable when targeting FGFR-dependent LC.

## Results

### Sensitivity to AZD4547 and BAY1163877 in LC cell lines

The antitumor activity of AZD4547 and BAY1163877, of which chemical structures were shown in Supplementary Figure 1a, was screened in 24 LC cell lines. Five of the twenty-four cell lines were reported to harbor *FGFR1* amplification (H1581, DMS114, H1703, H520 and HCC95).^21^ The full panel of LC cell lines, ranked from the most to the least sensitive to AZD4547 and BAY1163877 based on inhibition of cell proliferation, with the information of FGFR1 gene copy number and mRNA expression is shown in Supplementary Table 1. Of the 24 cell lines, 2 *FGFR1*-amplified LC cell lines, H1581 and DMS114, showed extreme sensitivity to AZD4547 and BAY1163877 (GI_50_ values ranging from 36 to 244 nm). All other cell lines were relatively resistant to AZD4547 and BAY1163877, with GI_50_ values ranging from 1.5 to >10 μm (Supplementary Table 1). H1581 and DMS114 showed sensitivity to FGFRi, whereas H1703, H520 and HCC95 showed resistance. Furthermore, H1581 and DMS114 cells do not have Met expression, whereas H1703, H520 and HCC95 cells do not show Met expression (Supplementary Figure 1b).

First, we tested whether there is any difference in the downstream signaling between sensitive (H1581 and DMS114) and resistant (H1703, H520 and HCC95) cell lines. Compared with the resistant cell lines, immunoblotting analysis of lysates from sensitive cell lines showed increased phosphorylation of FRS2α and phospholipase C (PLC)-γ, two major substrates of the FGFR kinase, but decreased phosphorylation of STAT3 (Supplementary Figure 1c). Overall, sensitive cell lines expressed high mRNA and protein level of FGFR1 in the presence of *FGFR1* amplification (Supplementary Table 1, Supplementary Figure 1c).

Importantly, phosphorylation of extracellular signal-regulated kinase (ERK)1/2 was significantly suppressed upon FGFR-TKI treatment in sensitive cells, suggesting that phosphorylated p-ERK1/2 may be a pharmacodynamic marker of downstream FGFR signaling (Supplementary Figure 1d). Phosphorylation of AKT is inhibited in DMS114, but not in H1581. In contrast, there was no effect of AZD4547 and BAY1163877 on p-ERK1/2 or p-AKT levels in the three resistant cells. Collectively, these data suggest that high basal level of FRS2α/PLC-γ and/or FGFR inhibition-induced ERK1/2 suppression may account for the sensitivity to AZD4547 or BAY1163877 in LC cells.

### Establishment of AZD4547- and BAY1163877-resistant cell lines derived from H1581 cells

**Figure 1:**
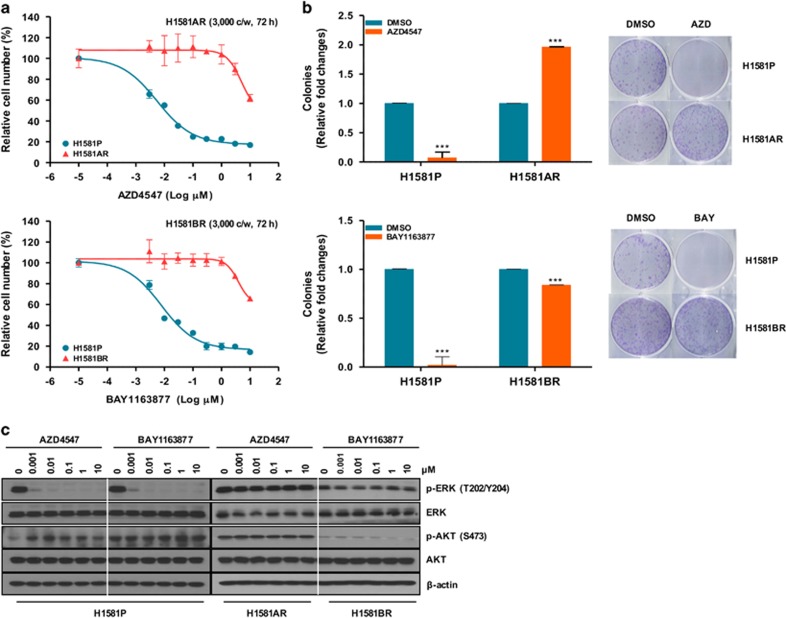
Establishment of H1581 cells resistant to the FGFR-TKIs AZD4547 and BAY1163877. (**a**) H1581P, H1581AR and H1581BR cells were treated with AZD4547 and BAY1163877 at the indicated concentrations, and viable cells were measured after 72 h of treatment. (**b**) Clonogenic assays of H1581P, H1581AR and H1581BR cells were treated with AZD4547 or BAY1163877 (1 μm) for 10 days. Data shown are the mean±s.d. of three independent trials. ***P<0.001; significantly different from vehicle control. (**c**) H1581P, H1581AR and H1581BR cells were treated with a dose escalation of AZD4547 and BAY1163877 for 2 h and cell extracts were immunoblotted to detect the indicated proteins. c/w, cells/well; DMSO, dimethyl sulfoxide; AZD, AZD4547; BAY, BAY1163877.

To explore the potential mechanisms of acquired resistance to selective FGFR-TKIs, we established drug-resistant cell lines by treating H1581 cells with stepwise increasing doses (up to 1 μm) of AZD4547 and BAY1163877 for ~6 months. We generated independent AZD4547- and BAY1163877-resistant cell lines derived from the highly-sensitive parental H1581 cells (H1581P). These resistant cell lines were designated H1581AR (AZD4547-resistant) and H1581BR (BAY1163877-resistant) cells, and subsequently maintained in media containing 1 μm AZD4547 or BAY1163877, respectively. AZD4547 and BAY1163877 dose-response curves of drug-resistant cell lines and H1581 cells confirm the acquisition of resistance to these drugs (Figures 1a and b). H1581AR cells exhibited higher resistance to AZD4547, with greater than 180-fold higher GI_50_ than H1581P cells (GI_50_=6.462±0.172 μm). H1581BR cells showed 100-fold higher resistance to BAY1163877 (GI_50_=3.986±0.050 μm). Consistent with these data, treatment with AZD4547 and BAY1163877 resulted in a significant decrease in colonies formed by H1581P cells, but not by H1581AR and BR cells (Figure 1b). Interestingly, remarkable morphological changes were noted in H1581AR and H1581BR cells. The spindle-like fibroblastic phenotype, characteristic of H1581P cells, was markedly attenuated in H1581AR and H1581BR cells, indicating loss of cell-to-cell connections in the resistant cell lines (Figure 1c). After treatment with AZD4547 or BAY1163877, no changes in p-ERK and p-AKT levels were detectable in H1581AR and BR cells (Figure 1d).

### Activation of Met in AZD4547- and BAY1163877-resistant cells

**Figure 2:**
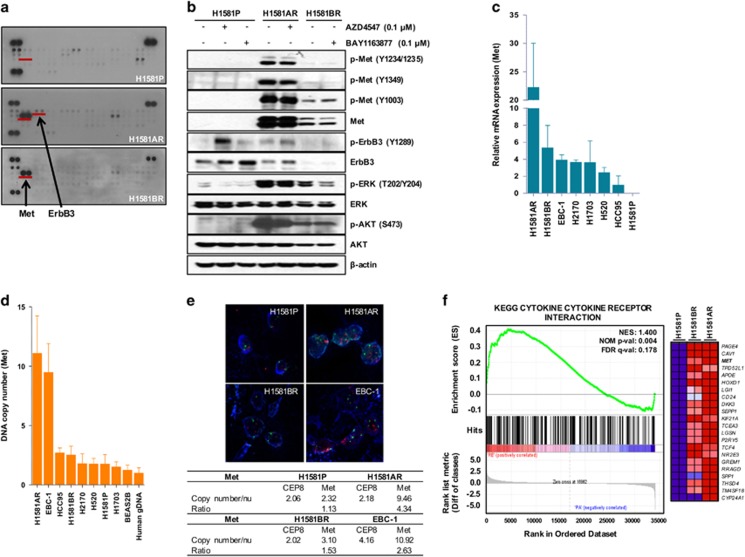
Met is upregulated in H1581AR and H1581BR cells. (**a**) p-RTK array analysis shows that H1581P, H1581AR and H1581BR cells maintain phosphorylation of Met in the presence of AZD4547 and BAY1163877. H1581P, H1581AR and H1581BR cell lysates were hybridized to a p-RTK array. Hybridization signals at the corners serve as controls. (**b**) H1581P and resistant cells were treated with 0.1 μm AZD4547 or BAY1163877 for 2 h. Cell extracts were immunoblotted to detect the indicated proteins. A Taqman assay was used to determine (**c**) mRNA expression and (**d**) DNA copy numbers of *MET*. (**e**) *MET* amplification in tumor cells as shown by FISH. There are increased copies of the orange red signal (probe binding to *MET*) compared with the green signal (CEP8, control chromosome enumeration probe). Data shown in (**c** and **d**) are the mean±s.d. of three independent trials. (**f**) A GSEA plot of KEGG cytokine–cytokine receptor interaction pathway showed significant enrichment in H1581AR and H1581BR vs H1581P (left panel). Heat map representation of the top 25 deregulated genes in H1581AR/BR cells vs H1581 (right panel). Previously identified gene sets (available at molecular signature database; MSigDB, C2; www.broadinstitute.org/gsea/) were screened to identify those that are differentially enriched in H1581AR/H1581BR cells vs H1581P by analyzing relative expressions using GSEA methods.^29^

Next, we sought to identify potential mechanisms of acquired resistance to FGFR-TKIs in H1581AR and H1581BR cells. Receptor tyrosine kinase (RTK) array and immunoblotting analyses demonstrated strong overexpression and activation of Met in H1581AR and H1581BR cells, compared with the nearly-undetectable expression in the parental cells (Figures 2a and b). The basal level of Met overexpression/activation in H1581BR cells was lower than that observed in H1581AR cells. Consistent with the RTK array data, ErbB3 phosphorylation was markedly elevated in H1581AR cells, compared with that in H1581P and H1581BR cells. In H1581AR and H1581BR cells, we also observed phosphorylated ERK and AKT, two downstream signaling transducers of the Met pathway (Figure 2b).

Next, we tested whether strong overexpression and activation of Met in H1581AR and H1581BR is associated with increased mRNA expression and/or gene copy number of Met. Here, we used the EBC-1 cell line, in which *MET* is reported to be amplified,^22^ as the positive control. All the resistant cells, including those with acquired (H1581AR, H1581BR) and intrinsic (H2170, H1703, H520, HCC95) resistance to FGFR-TKIs, expressed higher levels of Met mRNA, compared with that in H1581P cells (Figure 2c). Interestingly, despite Met activation, H1581BR cells did not harbor *MET* copy number gain (2.54) (Figure 2d). Consistent with quantitative real-time PCR results, H1581AR, but not H1581BR, showed marked *MET* gene amplification by fluorescence *in situ* hybridization (FISH) (>9 copies per cell; Figure 2e). These findings suggest that stronger Met overexpression/activation driven by *MET* amplification might result in higher level of drug resistance in H1581AR cells, compared with H1581BR cell (180-fold resistance to AZD4547 in H1581AR vs 100-fold resistance to BAY1163877 in H1581BR).

Next, H1581P, H1581AR and H1581BR cells were subjected to genome-wide gene expression profiling using cDNA microarray (Supplementary Table 2). The gene set enrichment analysis (GSEA) against Kyoto Encyclopedia of Genes and Genomes (KEGG) database (http://www.genome.jp/kegg/) identified four pathways (‘Axon guidance', ‘Adherens junction', ‘Focal adhesion' and ‘Cytokine–cytokine receptor interaction') that were significantly enriched, with Met contributing significantly to the core enrichment, in H1581AR and H1581BR cells, compared with H1581P cells (Figure 2f, Supplementary Tables 3 and 4).

**Figure 3:**
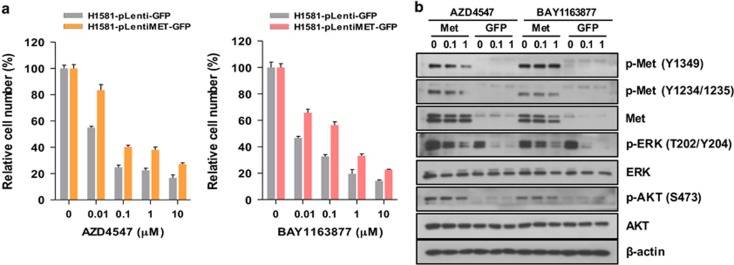
Overexpressed Met enhances resistance in H1581P cells. (**a**) H1581P cells were infected with lentivirus harboring a construct encoding GFP-tagged Met or the control plasmid carrying GFP alone. After lentivirus infection, cells were treated with the FGFR-TKIs at the indicated concentrations. Cells were infected as above with increasing amounts of lentivirus and treated with FGFR-TKIs. (**b**) Immunoblotting analysis for the indicated proteins.

To further examine the role of Met overexpression and activation in acquired resistance to FGFR-TKIs, we infected H1581P cells with lentivirus+ harboring a construct encoding GFP-tagged Met or the control plasmid carrying GFP alone. Ectopic expression of Met significantly induced resistance to AZD4547 and BAY1163877 in MTT assays (Figure 3a). Furthermore, Met overexpression induced activation of downstream ERK1/2 and AKT, which could not be abrogated by AZD4547 or BAY1163877 treatment (Figure 3b). Taken together, these data suggest that Met activation is responsible for the acquisition of acquired resistance to FGFR-TKIs in H1581P.

### Met-dependent ErbB3/PI3K signaling in H1581AR, but not in H1581BR cells

**Figure 4:**
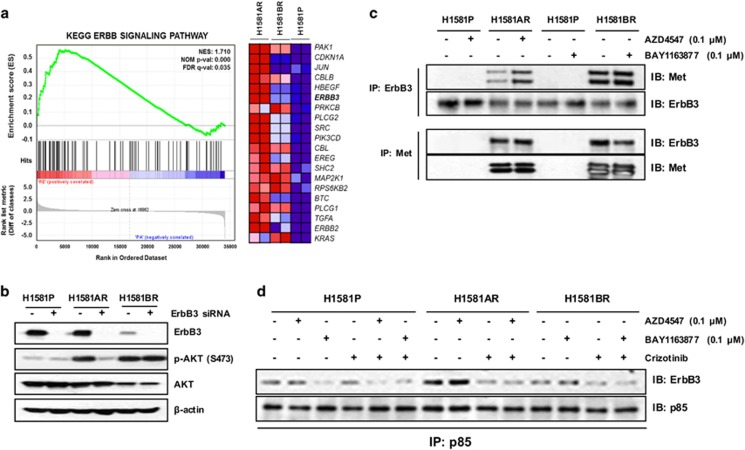
Activation of Met is a key determinant of mechanistic differences in difference types of FGFR-TKI-resistant cells. (**a**) GSEA plot showing the upregulated expression of previously known ErbB3-associated gene sets in H1581 acquired resistance cells, including genes that are commonly upregulated in H1581AR cell lines (‘KEGG_ERBB_SIGNALING_PATHWAY'). (**b**) H1581P, H1581AR and H1581BR cells were transfected with control siRNA or ErbB3-specific siRNA as described in Materials and methods. (**c**) Met/ErbB3 complex formation was evaluated by immunoprecipitation analysis. All cells were treated with 0.1 μm of each of the indicated FGFR-TKIs. (**d**) Met or ErbB3 and PI3K p85 subunit complex formation was evaluated using co-immunoprecipitation analysis. All cells were treated with 0.1 μm of each of the indicated FGFR-TKIs and 0.2 μm crizotinib. All cell extracts were immunoblotted to detect the indicated proteins.

*MET* amplification leads to gefitinib resistance in LC by activating ErbB3-dependent activation of PI3K.^17^ Given that ErbB3 phosphorylation was markedly elevated in *MET*-amplified H1581AR cells, but not in H1581BR cells (Figures 2a and b), we therefore hypothesized that acquired resistance to AZD4547 in H1581AR might involve sustained signaling via ErbB3. The GSEA of gene expression profiles revealed that the ERBB signaling pathway was significantly enriched, with ERBB3 contributing significantly to the core enrichment, in H1581AR cells, compared with H1581BR and H1581P cells (Figure 4a, Supplementary Table 5). Inhibition of Met by crizotinib, an ALK/MET TKI, or siMET fully suppressed ErbB3 and AKT phosphorylation, which suggests that Met can trigger the activation of ErbB3/PI3K signaling in H1581AR and H1581BR cells (Supplementary Figures 2a and b). Interestingly, downregulation of ErbB3 by an ErbB3-specific short interfering RNA (siRNA) led to substantial inhibition of AKT phosphorylation in H1581AR, but not in H1581BR cells, supporting the role of ErbB3 in mediating PI3K/AKT activation in *MET*-amplified H1581AR cells (Figure 4b). To confirm the validation findings for ErbB3, we treated H1581 cells with NRG1 (ligand to ErbB3) and H1581AR cells with AZD8931 (panHER inhibitor) and Met overexpressing cells (EBC-1 cell line) with ErbB3 siRNA (Supplementary Figures 2c–e). However, there was no change of resistance to FGFR-TKIs and unavailing. These findings also might suggest that there exist different mechanisms by which PI3K/AKT becomes activated in the two resistant cells.

To investigate how Met activates PI3K/AKT signaling in the two resistant cells, immunoprecipitation assays were performed. In H1581AR and H1581BR cells, ErbB3 coprecipitated with Met and vice versa and, as expected, both interactions were not disrupted by AZD4547 or BAY1163877 (Figure 4c). However, compared with H1581P and H1581BR, the association of ErbB3 with the p85 regulatory subunit of PI3K was greatly enhanced in H1581AR cells and vice versa (Figure 4d, Supplementary Figure 2c). The association of p85 with ErbB3 was effectively blocked by crizotinib or combination of crizotinib and AZD4547 in H1581AR cells. These observation suggest that Met may mediate transphosphorylation of ErbB3, which creates binding sites for PI3K, in H1581AR cells.

Taken together, these data suggest that *MET* amplification activates PI3K/AKT signaling through ErbB3 in H1581AR cells, whereas Met overexpression directly activates PI3K/AKT in H1581BR cells.

### Enhanced invasion, migration and EMT in H1581AR and H1581BR cells

**Figure 5:**
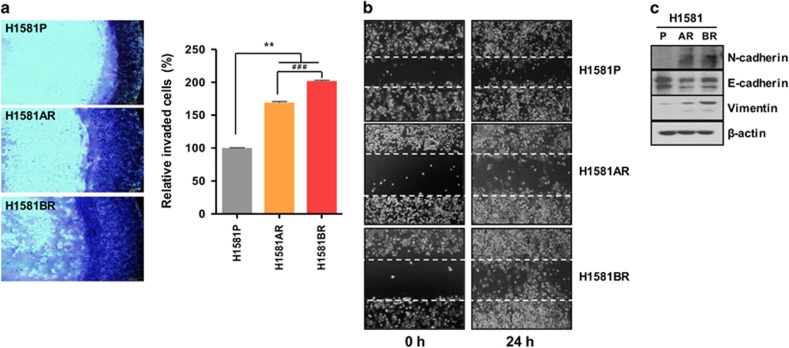
H1581-derived drug-resistant cells demonstrate metastatic phenotype. (**a**) Cell invasion assays (using QCM ECMatrix) were carried out. The percentages of invasion were quantitated by assessing the percentage of stained invasive cells. **P<0.01; ^###^*P*<0.001; significantly different from vehicle control. (**b**) *In vitro* scratch wound healing assay in H1581P, H1581AR and H1581BR cells. Representative images taken at the indicated time points post-wounding are shown. (**c**) H1581P, H1581AR and H1581BR cell extracts were immunoblotted to detect indicated proteins. Data shown in (**a**) are the mean±s.d. of three independent trials.

Given the fact that Met activation triggers enhanced invasion and metastasis of tumor cells,^23^ we further investigated whether constitutive Met activation in H1581AR and H1581BR cells resulted in upregulation of invasion and migration potentials of these resistant cells. H1581AR and H1581BR cells displayed significant enhancement of invasive and migratory potentials, compared with H1581P cells (Figures 5a and b). Immunoblotting analysis of the EMT markers indicated that H1581AR and H1581BR cells showed higher expression of N-cadherin and vimentin, but lower expression of E-cadherin, compared with the H1581P (Figure 5c). Taken together, our data suggest that acquisition of acquired resistance to FGFR-TKIs not only leads to morphological changes, but also promotes migration and invasion of cells, in part by inducing EMT.

### Combined inhibition of FGFR and Met signaling to overcome acquired resistance to FGFR-TKIs

**Figure 6:**
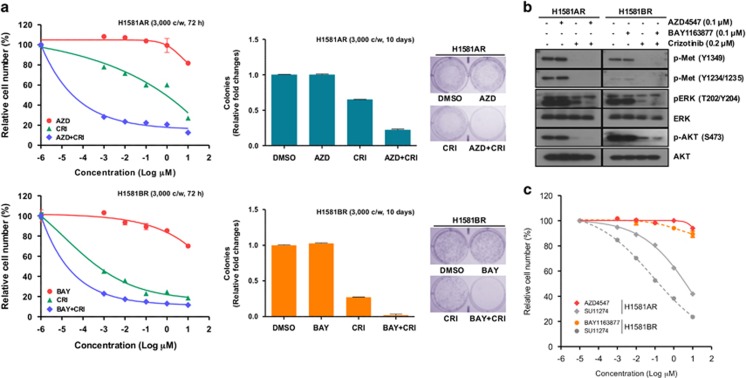
Inhibition of Met restores sensitivity to FGFR-TKIs. (**a**) H1581AR and H1581BR cells were treated with the indicated combinations of the FGFR-TKIs, AZD4547 (AZD) and BAY1163877 (BAY), along with crizotinib (CRI) for the indicated time and cell viability was analyzed using the MTT and clonogenic assays. (**b**) H1581AR and H1581BR cells were treated with either AZD4547 or BAY1163877 (0.1 μm) alone, or crizotinib (0.2 μm) alone or with the combination of AZD or BAY with crizotinib for 2 h. Cell extracts were immunoblotted to detect the indicated proteins. (**c**) H1581AR and H1581BR cells were treated with the indicated combinations of the Met inhibitor, SU11274, for the indicated time and cell viability was analyzed using the MTT assay.

To determine whether combined inhibition of Met signaling overcomes the acquired resistance to FGFR-TKIs, we tested the effects of combined treatment of crizotinib or siMET with FGFR-TKIs on cell viability. The combination of FGFR-TKIs with crizotinib or siMet synergistically inhibited cell proliferation in both H1581AR and H1581BR cells (Figure 6a, Supplementary Figure 3a). Similar data were seen in a clonogenic assay. Consistent with these data, combined inhibition of Met and FGFR signaling effectively suppressed ERK1/2 and AKT activation, compared with inhibition of either pathway alone (Figure 6b, Supplementary Figure 3b).

## Discussion

In our study, we evaluated *in vitro* antitumor activity and pharmacodynamic effects of selective FGFR-TKIs in a panel of LC. We found that two *FGFR1*-amplified LC cells with high mRNA and protein expression of FGFR1 were sensitive to FGFR-TKIs and pharmacodynamic inhibition of p-ERK1/2 may be responsible for the sensitivity to these agents. Importantly, we herein reported, for the first time, that enhanced Met signaling by gene amplification or overexpression confers acquired resistance to FGFR-TKIs. Notably, *MET* amplification-driven ErbB3 activation reactivated PI3K/AKT signaling, allowing H1581AR cells to bypass the growth-inhibitory effects of AZD4547. Our data provided preclinical evidence supporting a rationale for combined inhibition of FGFR and Met signaling to overcome acquired resistance to FGFR-TKIs for the treatment of LC.

*FGFR1* amplification has been considered as a novel druggable target in LC. Preclinical studies clearly demonstrated that *FGFR1* amplification confers dependence on FGFR signaling.^4^ Inhibition of FGFR1 in both *FGFR1*-amplified LC cell lines and xenograft models resulted in growth inhibition and apoptosis. These promising preclinical results provided the rationale for clinical studies of BGJ398 and AZD4547 for patients with *FGFR1*-amplified LC. However, the clinical results of these selective FGFR-TKIs were somewhat disappointing with overall responses of ~10%.^11^ Therefore, the therapeutic potential of FGFR inhibition in *FGFR1*-amplified LC will remain uncertain until more comprehensive understanding of FGFR pathway addiction is achieved.

The majority of LC cell lines tested in our study were resistant to AZD4547 and BAY1163877, presumably reflecting the recent disappointing clinical outcomes with selective FGFR-TKIs in LC patients. Notably, of the five *FGFR1*-amplified cell lines, only two (H1581 and DMS114) cell lines that express high mRNA and protein level of FGFR1 were sensitive to these agents. Recently, BGJ398-insensitive cells (NCI-H1703, HCC95 and Calu-3) were shown to express low mRNA and protein level of FGFR1 even in the presence of *FGFR1* amplification, indicting that both FGFR1 amplification and protein overexpression are required for the efficacy of FGFR-TKI in LC.^24^ Moreover, FGFR1 mRNA and protein expression, rather than *FGFR1* amplification, was also reported to predict sensitivity to FGFR-TKIs in LC and head and neck squamous cell carcinoma.^25, 26^

Resistance to FGFR-TKIs in *FGFR1*-amplified cells can be driven by multiple factors. First, *FGFR1* amplification is not necessarily associated with mRNA and protein overexpression of FGFR1, which, in turn, is more likely to activate FGFR pathway. For example, HCC95, a squamous cell LC cell line, showed low mRNA and protein expression of FGFR1 in the presence of *FGFR1* amplification at the genomic level (Supplementary Table 1). Second, there may be a marked genomic heterogeneity in the 8p11-12 *FGFR1* amplicon structure between sensitive (focal) *versus* resistant (broad) cells, and the influence of these differences on the degree of FGFR addiction is unknown.^6^ Finally, FGFR inhibitor-resistant H1703 cell line carrying a focal *FGFR1* amplification has been shown not to be dependent on FGFR1 but on amplified platelet-derived growth factor receptor-α.^21, 24, 27^ This observation suggests that co-occurrence of other activating oncogenes may relieve FGFR1 dependence, resulting in the primary resistance to FGFR inhibition. Moreover, our data show that is a correlation between Met expression and the response to the FGFR inhibitors, indicating that Met may have a role in *de novo* resistance to FGFR inhibitors (Supplementary Figure 1b, lower panel).

In our study, high basal level of FRS2/PLC-γ was observed only in FGFR inhibitor-sensitive cell lines. Upon ligand binding, subsequent downstream signaling occurs through two main pathways via the FRS2 and PLC-γ, leading ultimately to activation of MAPK and PI3K/AKT pathways.^8^ Similar to ours, an elevated level of FRS2 phosphorylation was observed in H1581 cells carrying focal *FGFR1* amplification, but not in cells harboring relatively broader levels of *FGFR1* amplification.^21^ Also, phosphorylation of ERK1/2 was inhibited only in FGFR inhibitor-sensitive cell lines, indicating that the MAPK pathway is the major signaling pathway engaged in FGFR-driven oncogenesis. Similarly, ERK1/2 phosphorylation was inhibited upon BGJ398 treatment in FGFR1 overexpressing BGJ398-sensitive cells.^24^

Little is known about the acquired resistance to FGFR-TKIs in LC. In a recent report, Chell *et al.*^28^ established a KMS-11 myeloma cell line carrying the FGFR3Y373C mutation with acquired resistance to AZ8010, an FGFR inhibitor, and their sequencing analysis of resistant cells revealed a point mutation at the gatekeeper residue in FGFR3 (FGFR3V555M). The other mechanism of acquired resistance, which involved ErbB2/3 activation in a rapid, reversible and ligand-dependent manner, was observed in a study of resistance to BGJ398 in the RT112 bladder cancer cell line.^29^ In this study, total and p-Met were undetectable in the BGJ398-resistant cells, in contrast to our findings of the exclusive activation of Met signaling mediating ErbB3-dependent or -independent activation of PI3K/AKT signaling seen in H1581AR and H1581BR cells.

Activation of alternative RTKs including Met mediates acquired resistance to targeted therapies in various types of cancers by maintaining the signaling of key downstream pathways.^14^ In our study, we examined the mechanisms underlying acquired resistance to an FGFR-TKIs and found Met overexpression in two different drug-resistant cell lines, only one of which had *MET* amplification. Compared with sensitive cells, acquired resistant cells (H1581AR and H1581BR) had overexpression and activation of Met. In cells with acquired resistance, cell proliferation underwent a dependency switch from predominantly the FGFR to the Met pathway, suggesting that Met signaling can compensate for FGFR inhibition. To the best of our knowledge, ours is the first report to date to demonstrate the association between Met overexpression and acquired resistance to the FGFR-TKIs in LC. Interestingly, quantitative PCR on genomic DNA and FISH analysis confirmed *MET* amplification in H1581AR, but not in H1581BR, cells. *MET* amplification led to acquired resistance to AZD4547 in H1581AR, through activation of ErbB3. Similar to our findings, *MET* amplification activates causes acquired resistance to gefitinib by activating EGFR-independent phosphorylation of ErbB3 in EGFR-mutant LC.^17^ We showed that active ErbB3 co-precipitates with p85 in a Met-dependent manner in *MET*-amplified H1581AR, but not in H1581BR, cells. We interpret the differences in the signaling complexes and cellular behavior of the two drug-resistant LC cell lines as an indication of the different cellular mechanisms subsumed to drive drug resistance, based on the level of *MET* amplification and/or Met overexpression. Furthermore, acquired TKI resistance was characterized by EMT, concomitant with Met activation. Importantly, combined treatment of FGFR-TKI with crizotinib or Met-specific siRNA synergistically inhibited cell proliferation in both H1581AR and H1581BR cells. As shown in Figure 6, the stronger activity of crizotinib is observed in BR cells, which do not show *MET* amplification/overexpression, whereas AZD-resistant cells that have *MET* amplification/expression show less sensitivity to crizotinib. We examined other commercially available Met inhibitor (SU11274) on the FGFR inhibitor-resistant cell lines (H1581AR or H1581BR) for cell viability analysis. Met inhibitor (SU11274) showed similar effect with that of crizotinib.

In conclusion, our findings suggest that Met activation is sufficient to bypass dependency on FGFR signaling and concurrent inhibition of these two pathways may be desirable to overcome acquired resistance to FGFR-TKIs in LC. Our data provide insight into a potential avenue for effective therapy of human cancers with acquired resistance to FGFR-targeted therapy.

## Materials and methods

### Cell culture and reagents

Twenty-four cell lines were purchased from American Type Culture Collection (ATCC). All cell lines were cultured in RPMI-1640; except H1581 cells were cultured in Dulbecco's Modified Eagle's Medium (DMEM) and Ham's F12 Nutrient Mixture (DME/F12; Invitrogen, Carlsbad, CA, USA). Human bronchial epithelial BEAS2B cells were cultured in supplemented keratinocyte growth medium (KGM) bullet kit medium (Lonza, Walkersville, MD, USA). AZD4547 and crizotinib were purchased from Sellekchem (Houston, TX, USA). BAY1163877 was a gift from Bayer Healthcare AG (Wuppertal, Germany)

### Generation of inhibitor-resistant cell lines

AZD4547-resistant (AR) and BAY1163877-resistant (BR) derivatives of H1581 cells were generated by exposing cells to stepwise increasing concentrations (up to 1 μm) of each inhibitor. During generation of resistant cell lines, H1581P cells were cultivated in the absence of DMSO. The stepwise exposure to increasing drug concentrations was carried out over ~4 months, after which time the IC_50_ concentrations were re-assessed in each resistant cell line. Cells were then maintained continuously in the presence of inhibitors at these new IC_50_ concentrations for a further 6 months.

### MTT assay

Cells (3000 cells/well) were seeded on 96-well plates at 37 °C. After overnight incubation, the cells were treated with the inhibitor for 72 h. Then, MTT reagent [3-(4,5-Dimethylthiazol-2-yl)-2,5-diphenyltetrazoliumbromide] was added to each well and incubated for 4 h at 37 °C. MTT solubilization solution/stop mix (Promega, Madison, WI, USA) was added to each well, mixed, and the plates were incubated overnight at 37 °C. After measuring the absorbance at 570 nm, the data were graphically displayed using GraphPad Prism version 5.0 for Windows (GraphPad Software Inc., San Diego, CA, USA).

### Clonogenic assay

Cells (3000 cells/well) were plated onto 6-well plates, incubated overnight at 37 °C, and then treated with indicated concentrations of inhibitor for 10 days. Colonies were fixed with paraformaldehyde (4%) and stained with crystal violet (0.05% w/v) for 30 min, after which time residual staining solution was removed and plates were washed with water.

### Immunoblotting and RTK array

Following treatment with drugs and/or siRNA, cells were lysed in lysis buffer (Cell Signaling Technologies, Beverly, MA, USA). Total cell lysates (20 μl/lane) were resolved by SDS–PAGE and blotted onto Immobilon-P membranes (Millipore, Volketswil, Switzerland). Primary antibodies specific for FGFR1, p-Met (Tyr1349), p-Met (Tyr1234/1235), Met, p-ErbB3 (Tyr1197), ErbB3, p-FRS2α (Tyr436), p-AKT (Ser473), AKT, p-ERK1/2 (T202/Y204), ERK1/2, p-STAT3 (Tyr705), STAT3, p-PLCγ (Tyr783), PLCγ, p-PKCδ (Ser660), PKCδ, E-cadherin and vimentin were purchased from Cell Signaling Technologies. Antibodies specific for FGFR2, FGFR3, FRS2, N-cadherin and β-actin were purchased from Santa Cruz (Santa Cruz, CA, USA). Human RTK arrays (R&D Systems, Minneapolis, MN, USA) were performed according to the manufacturer's instructions.

### Gene expression or GSEA

Quantitative analysis of RNA expression was performed using Illumina HumanHT-12v4 gene chip cDNA microarrays (Illumina, San Diego, CA, USA). Microarray data in the CEL format were normalized using the robust multiple array average normalization method. GSEA was performed using gene sets from the Molecular Signatures Database (MSigDB-C2 v4, Broad Institute, Cambridge, MO, USA).

### Quantitative RT–PCR

Total RNA was reverse-transcribed with the Superscript cDNA Synthesis Kit (Invitrogen). Real-time PCRs were carried out in triplicate for each sample using primers and TaqMan probes purchased from Applied Biosystems. Reactions were run on a StepOnePlus system (Applied Biosystems, Foster City, CA, USA) and were analyzed using the StepOne software version 2.3 (Applied Biosystems) for Windows. The relative expression level of each gene was determined by the ΔΔCt method. Expression of *MET* (Hs01565584_m1) was expressed relative to the mean of the endogenous control, GAPDH (Hs02758991_g1).

### MET copy number variation analysis

Total genomic DNA was purified from cells using gDNA MiniPrep kits (Intron, Seoul, Korea). Real-time PCRs were carried out in triplicate for each sample using primers and TaqMan probes purchased from Applied Biosystems. Reactions were run on a StepOnePlus system (Applied Biosystems) and were analyzed using the StepOne software version 2.3 for Windows. The relative expression level of each gene was determined by the ΔΔCt method. Genomic levels of *MET* (Hs05018546_cn) were expressed relative to the mean of the endogenous control, LINE1 (Hs01098704_cn).

### FISH analysis

FISH assay was performed using *MET*-probe with Spectrum Orange (red) and CEP8 with Spectrum Green (Abbott Molecular, Des Plaines, IL, USA) following routine methods. FISH patterns were defined and described in previous studies.^4^ Cases were considered as *MET* positive (‘amplified') under one of the following conditions: the *MET*/CEP8 ratio ⩾2.0; the average number of *MET* signals per tumor cell nucleus is ⩾6; the percentage of tumor cells containing ⩾15 *MET* signals or large clusters is ⩾10% or the percentage of tumor cells containing ⩾5 *MET* signals is ⩾50%.

### Transfection of siRNAs

siRNAs against Met were synthesized by IDT (the sequence of siRNA listed in Supplementary Table 6) and a control siRNA was purchased from Invitrogen. Cells were transfected with siRNA (10 nm final concentration) for 48 h using Lipofectamine RNAiMax (Invitrogen) according to the manufacturer's instructions and then treated with the indicated drugs.

### MET overexpression by lentiviral infection

H1581 cells overexpressing *MET* were established by lentiviral infection and selection. 293FT cells were co-transfected using Lipofectamine 2000 (Life Technologies, Gaithersburg, MD, USA) with lentivirus packaging plasmid and a *MET* expression plasmid (Addgene 37560) or pLenti CMV GFP Puro (Addgene 17448). Forty-eight hours after transfection, viral supernatants were collected for viral infection, and infected H1581 cells were selected using puromycin (1 μg/ml) for 2 days.^30^

### Immunoprecipitation

For the detection of Met/ErbB3 complexes, whole-cell lysates (1 mg) in cell lysis buffer (Cell Signaling) were incubated with Protein A/G PLUS-Agarose beads pre-conjugated with Met or ErbB3. Immunoprecipitates were washed in lysis buffer, boiled in 2 × IP sample buffer, and analyzed by immunoblotting using antibodies specific to the indicated proteins.

### Statistical analysis

All numeric values are represented as the mean±s.d. Statistical significance of the data was determined using the Student's unpaired *t*-test. Values of *P*⩽0.05 were considered as statistically significant.
