# Supplementary material for: Activation of the Met kinase confers acquired drug resistance in FGFR-targeted lung cancer therapy
Source: Oncogenesis. 2016 Jul 18;5(7):e241–. doi: 10.1038/oncsis.2016.48 (PMC5399172; doi:10.1038/oncsis.2016.48)
Supplement: Supplementary Figure Legends [file oncsis201648x1.docx]

**Supplementary Figures**

**Supplementary Figure 1. Efficacy of the FGFR-TKIs on various lung cancer cell lines.** (a) Chemical structure of AZD4547 or BAY1163877. (b) Five lung cancer cell lines were treated with a dose escalation of AZD4547 or BAY1163877 for 72 hours (upper panel) and cell extracts were immunoblotted to detect the Met protein (lower panel). (c) Five lung cancer cell lines extracts were immunoblotted to detect the indicated proteins. (d) Five lung cancer cell lines were treated with a dose escalation of AZD4547 or BAY1163877 for 2 hours. Cell extracts were immunoblotted to detect the indicated proteins.

**Supplementary Figure 2.** **Role of Met and ErbB3 on H1581AR and H1581BR cells.** (a) H1581AR and H1581BR cells were treated with 1 μM crizotinib for 2 hours. (b) H1581AR and H1581BR cells were transfected with control siRNA or Met-specific siRNA as described in Materials and Methods. All cell extracts were immunoblotted to detect the indicated proteins. (c) H1581P cells were co-treated with NRG1 (20 ng/ml) and 0.1 µM of AZD4547 or BAY1163877 for 72 hours. Cell viability was analyzed using the MTT assay. Data shown are the mean ± SD of three independent trials (n.s.; no significant, ***; *p < 0.001*). (d) H1581AR and H1581BR cells were transfected with control siRNA or ErbB3-specific siRNA as described in Materials and Methods. (e) H1581AR cells were treated with AZD8931 for 72 hours. Cell viability was analyzed using the MTT assay. (f) Met or ErbB3 and PI3K p85 complex formation was evaluated using co–immunoprecipitation analysis. All cells were treated with 0.1 µM of each of the indicated FGFR-TKIs and 1 µM crizotinib. Cell extracts were immunoblotted to detect the indicated proteins.

**Supplementary Figure 3.** **Inhibition of Met restores sensitivity to FGFR TKIs in drug resistant cells.** (a) H1581AR and H1581BR cells were transfected with control siRNA or Met-specific siRNA as described in Materials and Methods. Cells were treated with FGFR-TKI (0.1 µM) for 72 hours and cell viability was analyzed using the MTT assay. (b) H1581AR and H1581BR cells were transfected with control siRNA or Met-specific siRNA as described in Materials and Methods. Forty-eight hours after transfection, cells were treated with FGFR-TKI (0.1 µM) for 2 hours. Cell extracts were immunoblotted to detect the indicated proteins. c/w, cells/well; DMSO, dimethyl sulfoxide; AZD, AZD4547; BAY, BAY1163877.
