# Supplementary figures and images for: Activation of the Met kinase confers acquired drug resistance in FGFR-targeted lung cancer therapy
Source: Oncogenesis. 2016 Jul 18;5(7):e241–. doi: 10.1038/oncsis.2016.48 (PMC5399172; doi:10.1038/oncsis.2016.48)

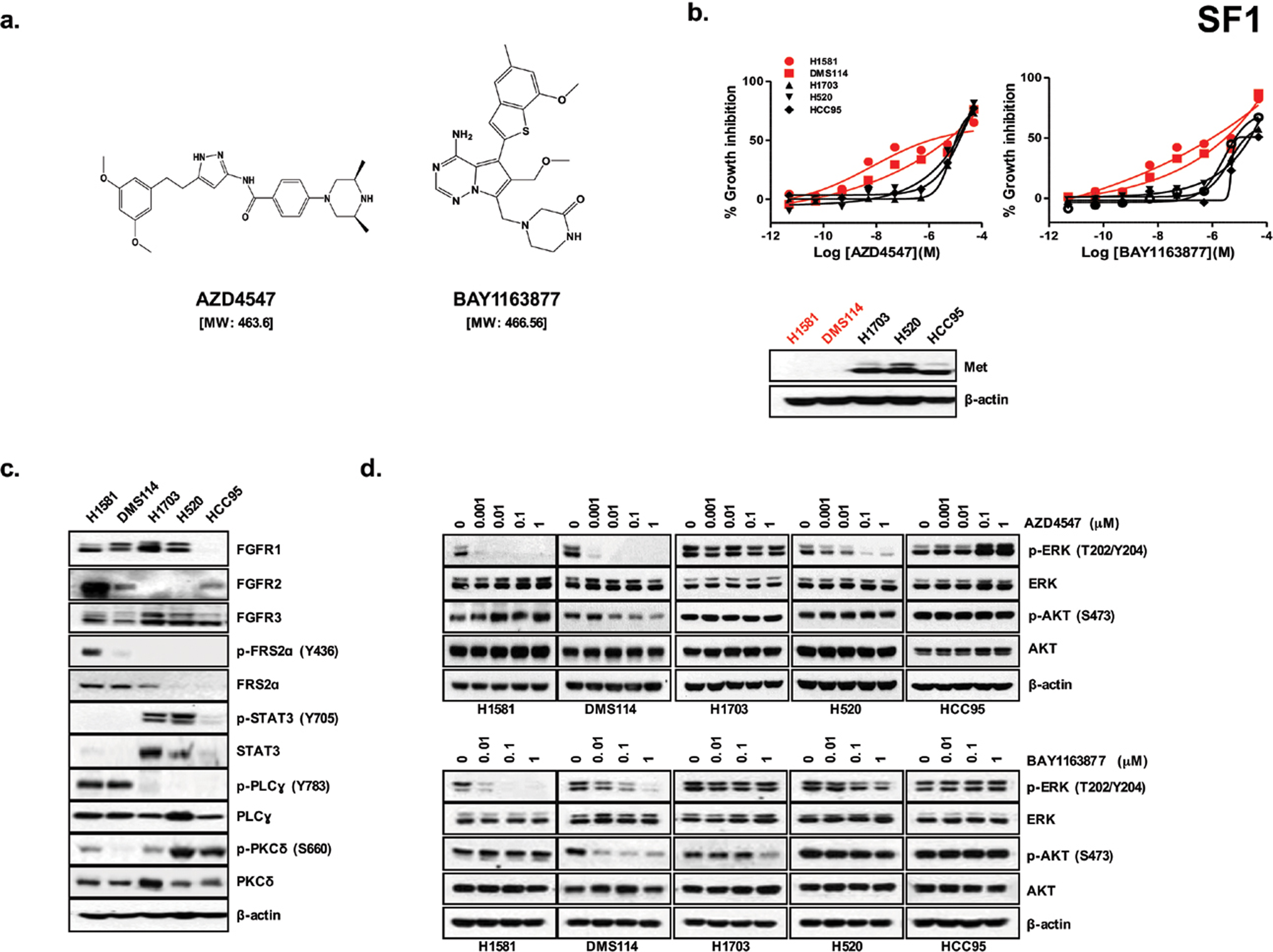

Supplement: Supplementary Figure 1 [file oncsis201648x2.tif]

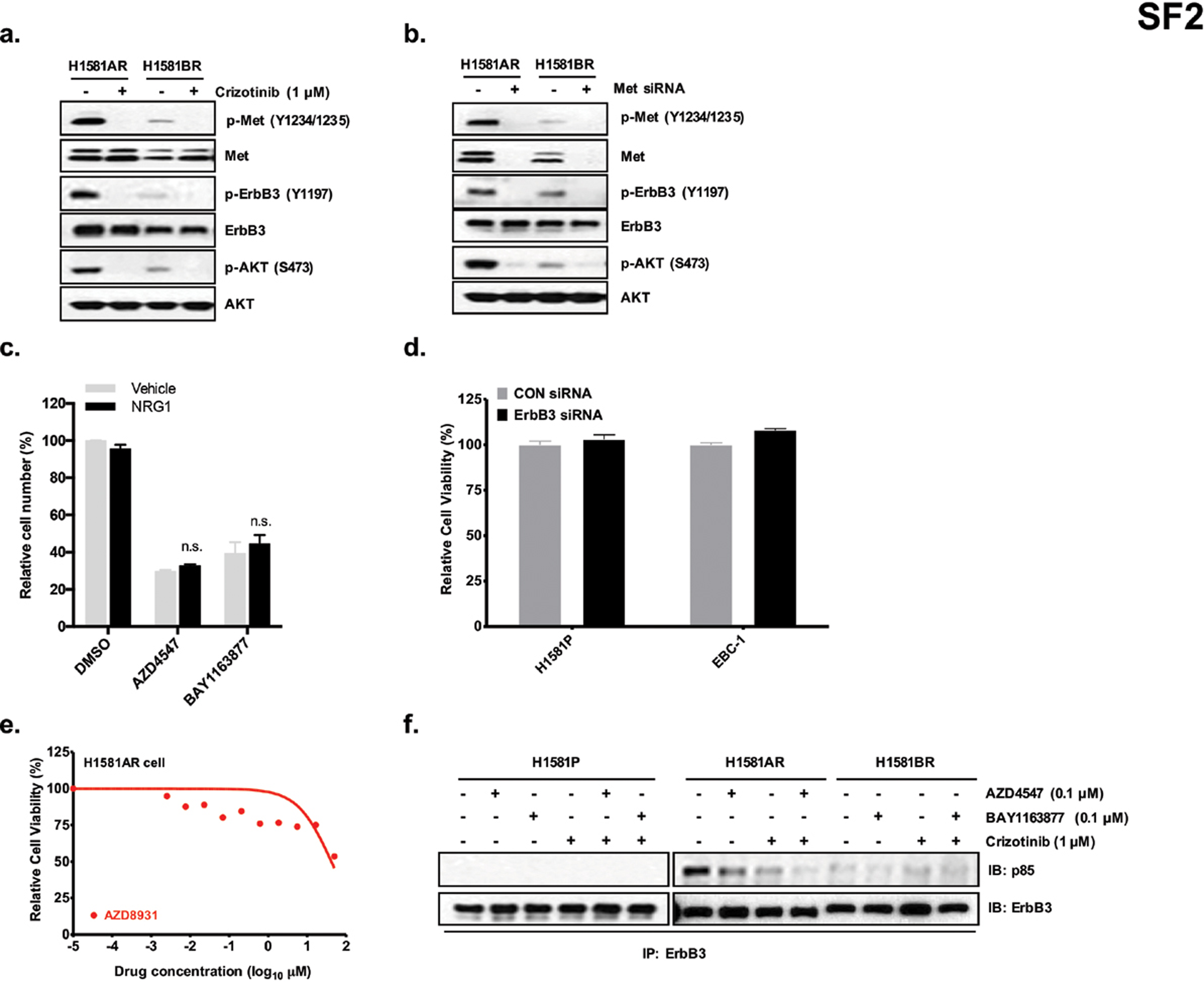

Supplement: Supplementary Figure 2 [file oncsis201648x3.tif]

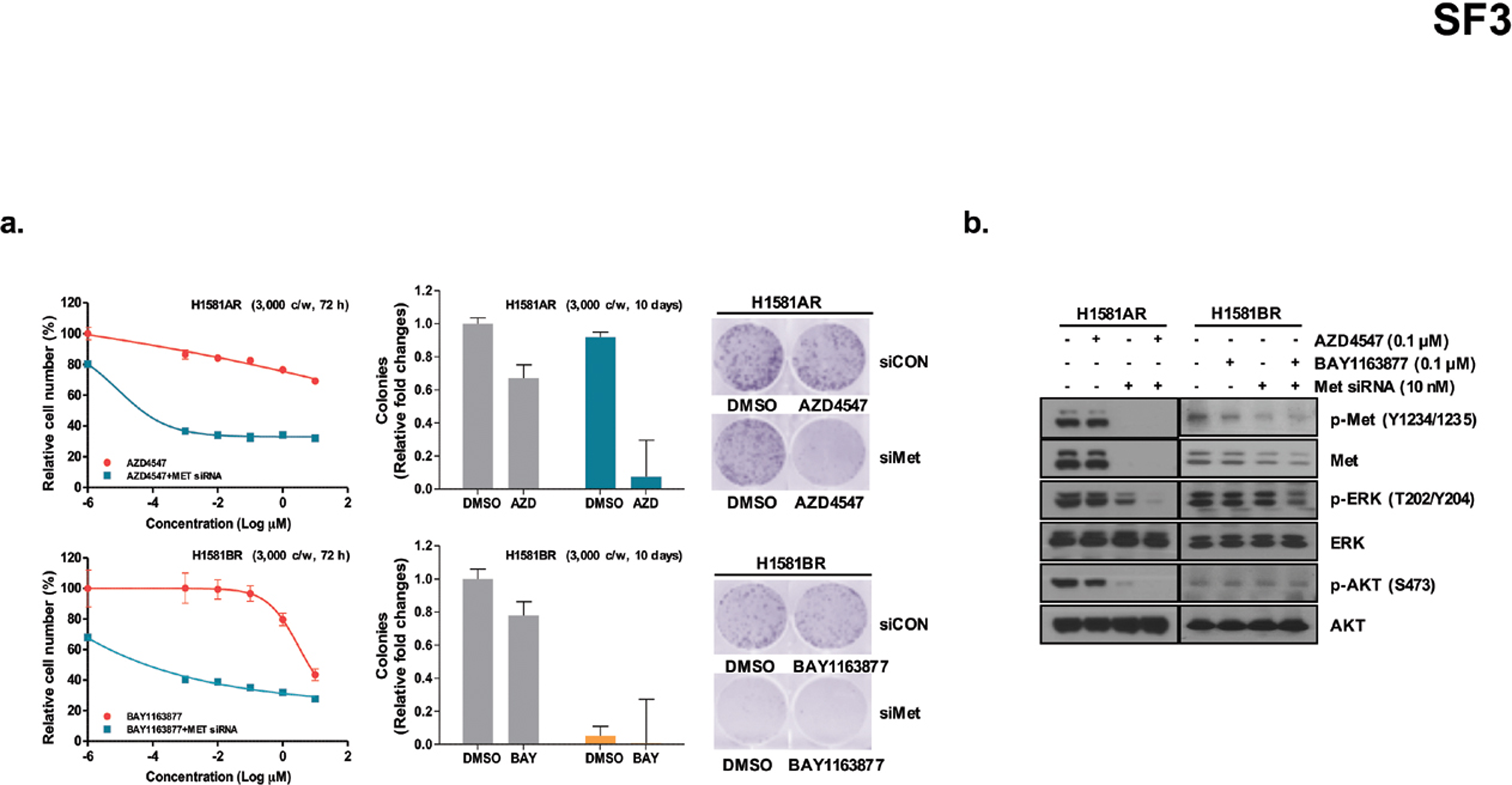

Supplement: Supplementary Figure 3 [file oncsis201648x4.tif]
